# Association Between Eye Color and Postoperative Pain in Female Patients With Symptomatic Irreversible Pulpitis in Mandibular Molars: A Prospective, Parallel-Group, Observational Study

**DOI:** 10.1155/2024/8824366

**Published:** 2024-10-23

**Authors:** Merve Sarı, Koray Yılmaz

**Affiliations:** Department of Endodontics, Faculty of Dentistry, Hatay Mustafa Kemal University, Hatay, Turkey

**Keywords:** eye color, inferior alveolar nerve block, irreversible pulpitis, pain, phenotype

## Abstract

**Background:** Current evidence indicates that some phenotypic characteristics, such as eye or hair color, might be associated with the experience of pain. We, therefore, compared the anesthetic success rate of inferior alveolar nerve block (IANB) and postoperative pain scores between light eyes and dark eyes in female patients who experienced symptomatic irreversible pulpitis (SIP) in a mandibular molar.

**Methods:** This prospective, parallel-group, observational study was registered with ClinicalTrials.gov (NCT06206304). A total of 110 adult female patients who experienced moderate or severe pain with SIP participated in this study. All patients received IANB with 4% articaine with 1:100.000 epinephrine. Endodontic access cavity preparation was initiated after confirmation of IANB. Pain during treatment was recorded by using a Visual Analog Scale. Anesthetic success was recorded as “none” or “mild” pain. Root canal treatment was performed, with standardized protocols. Postoperative pain scores were also recorded at 24, 48, and 72 h and 7 days after treatment. Statistical analyses of the data were performed using the independent *t*-test, repeated measures ANOVA test, and Pearson's chi-square test. The statistical significance level was set at 0.05.

**Results:** No significant differences were found in the success rate of IANB and postoperative pain scores between light- and dark-eyed patients at any time point (*p* > 0.05). The success rate of IANB was 72.73% and 67.27% for light- and dark-eyed patients, respectively.

**Conclusion:** Pain scores decreased significantly after RCT in both groups on all days (*p* < 0.05). No significant differences were found in the success rate of IANB and postop pain scores between light- and dark-eyed female patients who experienced SIP in a mandibular molar.

**Trial Registration:** ClinicalTrials.gov identifier: NCT06206304

## 1. Introduction

The International Association for the Study of Pain defined pain as “an unpleasant sensory and emotional experience associated with actual or potential tissue damage,” highlighting its individualized nature [1]. Pain perception involves a complex process of peripheral signaling, central processing, cortical activation, and behavioral response [2]. This neural activity, triggered by tissue damage, can be influenced by physiological, environmental, and genetic factors, leading to varied responses among individuals [3].

Research suggests a potential correlation between phenotypic characteristics expressed by pain-related genes and individuals' pain perception [4]. For example, individuals with red hair require higher desflurane concentrations for general anesthesia [5], are more resistant to the analgesic efficacy of lidocaine, and are more sensitive to thermal pain [6]. Similarly, a more recent study reported that red-haired women are more sensitive to pain [7]. This relationship is explained by the fact that the red hair phenotype is induced by a mutation in the melanocortin-1-receptor (MC1R) gene, and MC1R gene receptors are expressed in the mesencephalic periaqueductal gray, one of the important points in the pain pathway [8]. Droll et al. [9] reported that the level of dental anxiety was greater among women with red hair and mutations in the MC1R gene; nevertheless, there was no significant difference in the success of inferior alveolar nerve block (IANB) with lidocaine between red-haired and dark-haired women.

The eye color phenotype may be associated with pain perception, as is the case with hair color [10]. The etiology of human pigmentation is based on complex gene–gene and gene–environment interactions [11]. Oculocutaneous albinism (OCA) is characterized by light hair color and sun-sensitive skin due to reduced melanin synthesis. It is associated with mutations in the human P gene, also known as OCA2. Gene–gene interactions between OCA2 and MC1R have been found to have a significant effect on skin, hair, and eye color pigmentation [12, 13]. However, few studies have considered eye color when evaluating the relationship between pigmentation-related phenotypic characteristics and pain perception [10, 14].

Previous studies reported that it is challenging to achieve adequate pulpal anesthesia in mandibular molar teeth with symptomatic irreversible pulpitis (SIP), and the success rate of IANB ranges between 23% and 93% [15–18]. A possible explanation for this high IANB failure rate is the release of inflammatory mediators and proinflammatory neuropeptides due to pulpal inflammation. Inflammatory mediators increase pain sensation and neuronal excitability by lowering the activation threshold of peripheral nociceptors [19].

The prevalence of postoperative pain after root canal treatment (RCT) is between 3% and 58 [20]. Postoperative pain is multifactorial. It might be affected by host-related factors or chemical, mechanical, or microbiological injury associated with endodontic procedures [21]. Extrusion of debris, irrigation solutions, intracanal medications, filling materials, and their components can come into contact with periradicular tissues, potentially triggering postoperative pain by increasing the chemotaxis of inflammatory cells such as histamine, prostaglandins, and neuropeptides [22, 23]. Despite the investigation of these factors, the association between phenotypic characteristics and postoperative pain following RCT has not yet been evaluated.

Identifying the phenotypic characteristics associated with higher pain sensitivity may benefit clinicians in managing postoperative pain, which is an undesirable experience for both the patient and the practitioner. We, therefore, aimed to assess the anesthetic success rate of IANB and postoperative pain scores between light- and dark-eyed female patients experiencing SIP in a mandibular molar. The null hypothesis was that eye color has no effect on the IANB anesthetic success rate or postoperative pain scores.

## 2. Materials and Methods

This prospective, parallel-group, observational study was approved by the Ethics Committee and registered with ClinicalTrials.gov.

### 2.1. Sample Size Determination

The sample size was determined according to a previous study [24], and a minimum of 51 subjects were included in each group (effect size = 0.5, *α* = 0.05, and power *β* = 0.80). Patients admitted to the Hatay Mustafa Kemal University Faculty of Dentistry Endodontics Clinic for root canal treatment from June 2023 to December 2023 were evaluated and 110 subjects were included in this study, *n* = 55 for each group (1:1 ratio).

### 2.2. Inclusion Criteria

Female patients (gender definition is based on patient self-reports) with no systemic diseases (ASA 1–2); aged between 18 and 45 years; eye colors of brown, hazel, green, or blue; and mandibular molar teeth diagnosed with SIP were included in this study. The diagnosis of SIP was confirmed by a prolonged response to a cold test (Endo Ice, Coltene, Altstatten, Switzerland) and responsiveness to the electric pulp test. Patients with moderate (4–6 mm) or severe pain (7–10 mm) according to the Visual Analog Scale (VAS) were included. Teeth with no periodontal problems (probing depth ≤ 3 mm) and without periapical radiolucency were included.

Each patient was informed, and written consent was obtained to participate in the study.

### 2.3. Exclusion Criteria

The exclusion criteria were as follows: male sex; use of colored contact lenses; surgical operation to change the iris color; neurophysiological disease; use of medications such as antidepressants or nonsteroidal anti-inflammatory or opioid drugs; and pregnancy.

### 2.4. Categorization of the Patient's Eye Color

Categorization of the patient's eye color was performed by an independent researcher. The eye color of all patients was examined at the same point under the same lighting conditions. Patients were also asked to indicate their own eye color. If a consensus could not be reached between the researcher and the patient regarding eye color, the patient was excluded from the study. The blue-green color was categorized as light; all shades of brown color were categorized as dark [25].

### 2.5. Root Canal Treatment Procedures

Patients' anxiety ratings were determined using the Modified Dental Anxiety Scale (MDAS) by a blinded operator before treatment. The anxiety scale consists of five questions, each of which can be scored from 1 to 5 (from least to most) [26]. Considering the effect of circadian rhythm on pain sensitivity [27], treatment time was limited and all treatment procedures were performed in the morning (9:30–12.30 a.m.). All dental procedures, including anesthesia injection, were performed by a single endodontist. A total of 119 patients received IANB injections of 1.8 mL 4% articaine (1:100.000; Ultracain D-S) using the Halstead method. A 27-gauge needle (Beybi Medical Co) was used for all the injections. The anesthetic solution was slowly deposited into the target area for 60 s. Ten minutes after IANB anesthesia, patients were asked whether their lips were numb. Patients who reported no profound lip numbness were excluded. In patients who reported lip numbness, anesthesia was confirmed with a pulp sensibility test. A traditional access cavity was prepared under an abundant water coolant after rubber dam isolation. Patients were instructed to inform the operator if they felt pain during access cavity preparation. Pain intensity was rated into four categories using the VAS as follows:

1. .No pain (0)
2. .Mild pain (1–3 mm)
3. .Moderate pain (4–6 mm)
4. .Severe pain (7–10 mm).

IANB anesthesia was defined as successful when the patient reported no pain or mild pain during access cavity preparation. IANB anesthesia was considered a failure in patients with moderate or severe pain; intrapulpal anesthesia was administered in these cases.

After the canal orifices were located, the working length (WL) was determined using an apex locator (Morita Root ZX) and confirmed by radiographs. Root canals were prepared using the R25 (Reciproc, VDW) file in the mesial root canals and the R25, R40, and R50 files in the distal root canals, respectively, with the crown-down technique. A total volume of 20 mL of 2.5% sodium hypochlorite (NaOCl) was used for each canal with a 30G needle (Max-i-Probe; Dentsply). The final irrigation was performed with 3 mL of 17% ethylenediaminetetraacetic acid (EDTA; Coltene) solution. Endoactivator (EA; Dentsply) 25/04 tip placed 2 mm behind the WL was used in 3 cycles of 20 s each for EDTA. Five milliliters of distilled water was used between the solutions and the root canals were dried with paper points (VDW). The root canals were filled with lateral condensation using gutta-percha cones (VDW) and an epoxy-resin-based sealer (AH Plus; Dentsply) in a single visit. All teeth were restored with resin composite (Estelıte Sıgma Quıck; Tokuyama). The root canal filling was checked by radiographs.

The patients were prescribed 400 mg of ibuprofen and instructed to take it in the presence of pain. Patients recorded their pain on days 1, 2, 3, and 7 after treatment according to the VAS scale. Pain scores and analgesic usages were recorded by an independent researcher.

### 2.6. Statistical Analysis

Statistical analysis of the data was performed using a software program (IBM SPSS Statistics, v22; IBM Corp). The data were normally distributed according to the Shapiro–Wilk normality test. A between-group comparison for the light-eyed and dark-eyed patients for age was performed using the independent samples *t*-test. The independent samples *t*-test was used to compare pain scores and anxiety scores between light- and dark-eyed patients. The repeated measures ANOVA test was used to evaluate the changes in pain scores over time. Pearson's chi-square test was used to analyze anesthetic success rates and analgesic intake. The results are expressed as 95% confidence intervals (95% CIs). The statistical significance level was set at 0.05.

## 3. Results

**Figure 1:**
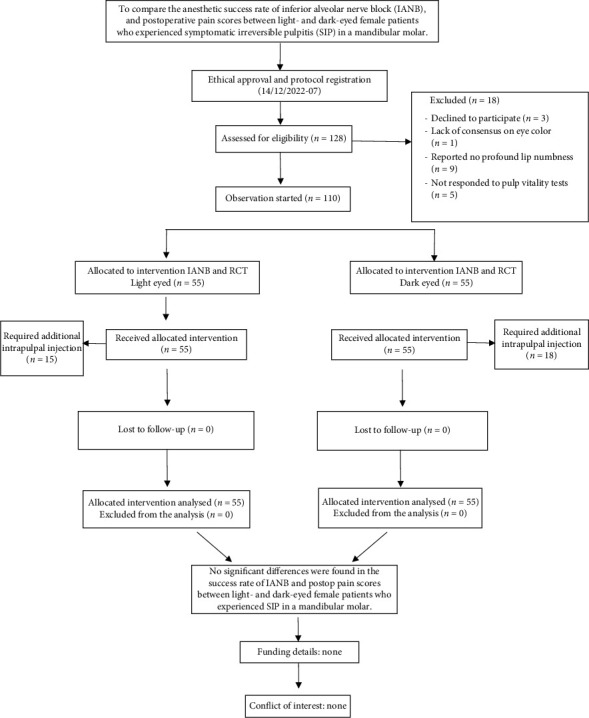
Flowchart of the participants throughout the trial.

Among the 128 patients assessed for the study, 110 patients met the inclusion criteria. The reasons for exclusion were declining to participate in the study (*n* = 3), lack of consensus on eye color (*n* = 1), failure to achieve profound lip numbness (*n* = 9), and not responding to pulp vitality tests (*n* = 5). A total of 110 patients (*n* = 55) underwent root canal treatment and there was no dropout during the follow-up (Figure 1). The mean MDAS score for light- and dark-eyed patients is 10.71 ± 3.804 and 9.37 ± 3.235, respectively, with no significant difference (*p* > 0.05) (Table 1). The median anxiety rating was 2 for both groups.  Table 2 shows the mean preoperative and postoperative pain scores. The preoperative pain scores were 6.32 ± 0.18 and 6.71 ± 0.32 for light eyes and dark eyes, respectively, and were comparable between the two groups (*p* > 0.05). Postoperative pain scores decreased significantly in both groups on all days (*p* < 0.05); nevertheless, no significant differences were found between the two groups at any time point evaluated (*p* > 0.05). The success rate of IANB was 72.73% and 67.27% for light eyes and dark eyes, respectively (*p* > 0.05). There was no significant difference in analgesic intake between the two groups (*p* > 0.05). The mean age was 34.4 ± 9.9 and 31.3 ± 9.1 years for the light eyes and dark eyes, respectively, and was comparable between the two groups (*p* > 0.051) (Table 3).

## 4. Discussion

The role of genetic factors in pain mechanisms and responses to analgesic drugs has been widely investigated, and genetic factors are heritable [28]. Unraveling the relationship between phenotypic characteristics and pain experience might be beneficial for endodontic pain management. Therefore, in this prospective, parallel-group study, we compared IANB anesthetic success rates and postoperative pain scores between light- and dark-eyed female patients who experienced SIP in a mandibular molar. No significant differences were found in either IANB success rates or postoperative pain scores, and thus the null hypothesis was accepted.

Epidemiologic studies have shown that women are more sensitive to painful stimuli [29], require more analgesic medication [30], and suffer from chronic painful conditions such as migraine and fibromyalgia more frequently than men [31]. In this study, only female patients were included to reduce the variable effect of sex.

Dental anxiety can be defined as an emotional state of anxiety in response to the feared stimulus of dental treatment. Preoperative anxiety has been shown to be associated with higher anesthesia levels [32] and severe postoperative pain [33]. In this study, the MDAS was used to evaluate preoperative anxiety levels due to its ease of use and the short time duration of completion. The results revealed slight anxiety levels in both groups, with no significant differences between the groups.

Only patients with moderate or severe pain according to the VAS were included in this study. In previous studies, patients who experienced preoperative pain, female patients, and mandibular molar teeth were associated with higher postoperative pain [34–36]. A strong correlation has also been reported between high preoperative pain and intraoperative pain [37]. In this study, IANB anesthesia was performed with 4% articaine. Articaine has a longer anesthesia duration and provides better diffusion into the bone because of its high lipophilicity [38]. In this study, although a higher anesthesia success rate was observed in light-eyed patients (72.73%) than that in dark-eyed patients (67.27%), the difference was not statistically significant.

In this study, similar to literature [20], the high preoperative pain intensity significantly decreased within 24–48 h, following the treatment and decreased to minimum levels by the end of the first week. However, postoperative pain scores did not differ significantly between light- and dark-eyed patients at any time point (*p* > 0.05). Few studies have investigated the relationship between eye color and pain experience. In a pilot study involving a population of women in labor, Teng and Belfer [25] reported that compared with light-eyed individuals, dark-eyed individuals presented increased levels of anxiety and a tendency to experience more pain both at rest and during movement after receiving epidural analgesia. Holmgaard et al. [39] used pressure pain thresholds (PPTs) and the cold pressor test (CPT) to examine the relationships between phenotypic characteristics such as eye and hair color and pain perception. No significant difference was found between light- and dark-eyed individuals for PPT, while dark-eyed individuals were more sensitive to CPT than light-eyed individuals were.

Sutton [10] was the first researcher to investigate the relationship between eye color and pain in dentistry. The author reported that as the color of the iris changed from light to dark brown, greater pain reactions were observed during cavity preparation. A recent study reported that there was no difference between light- and dark-eyed women in terms of pain levels experienced during the insertion of the dental injection needle into the tissue and the administration of the anesthetic solution for infiltration anesthesia of the maxillary lateral incisor [14].

Although the narrative review highlighted that red-haired women have increased sensitivity to pain and reduced analgesic response [40], Myles, Buchanan, and Bain [41] and Gradwohl et al. [42] reached contrary findings. They reported that a patient's natural hair color does not affect anesthesia requirements, the speed of anesthesia, or the quality of postoperative recovery. Intraoperative factors, as in surgical procedures, can significantly impact postoperative pain following RCT. In this study, the fact that all treatments were performed by a single endodontist and that root canal preparation, irrigation, and obturation procedures were standardized could explain the lack of differences between the groups.

Pain is a subjective experience influenced by various biological, psychosocial, and genetic factors. One of the limitations of this study is the difficulty in accurately identifying pain, which is a multifactorial phenomenon. Another limitation of this study is that the OCA2 and MC1R genes, which are related to the subject, have not been investigated in the patients. Previous studies have identified genes associated with pain perception or analgesia [43, 44]. While an apparent genetic association will not always result in an absolute phenotypic effect, elucidating the biological mechanisms between pain genes and pigmentation genes may allow the development of individualized approaches to pain management. However, further research with larger populations and genetic testing is needed to explore and validate this relationship.

Within the limitations of this study, high preoperative pain scores decreased significantly after RCT in both groups. Eye color did not affect the result; no significant differences were found in the success rate of IANB and postoperative pain scores between light- and dark-eyed female patients experiencing SIP in a mandibular molar. More studies are needed to determine the clinical benefit of eye color in predicting the likelihood of experiencing pain related to RCT.

## Figures and Tables

**Table 1:** Preoperative MDAS scores of the light- and dark-eyed patients.

	Mean ± SD	Total score (min-max)	95% CI
Light eyed	10.71 ± 3.804	(5–17)	(−1047)–(3722)
Dark eyed	9.37 ± 3.235	(6–17)	(−1076)–(3751)
Sig. (2-tailed)⁣^∗^	0.267		

Abbreviations: CI, confidence interval; SD, standard deviation.

⁣^∗^*t* test.

**Table 2:** Preoperative and postoperative pain scores of the light- and dark-eyed patients.

Time period	Mean ± SD		*p* value
	Light eyed	Dark eyed	
Preop	6.71 ± 0.32^Aa^	6.32 ± 0.18^Aa^	0.31⁣^∗^
Postop 24th hour	1.63 ± 1.26 ^Ba^	1.88 ± 1.49^Ba^	0.59⁣^∗^
Postop 48th hour	0.89 ± 1.29 ^Ca^	1.12 ± 0.99^Ca^	0.29⁣^∗^
Postop 72nd hour	0.42 ± 0.77 ^Da^	0.47 ± 0.80^Da^	0.82⁣^∗^
Postop 1st week	0.16 ± 0.38 ^Da^	0.24 ± 0.44^Da^	0.56⁣^∗^
	*p* < 0.001^*r*^	*p* < 0.001^*r*^	

*Note:* Different superscript capital letters indicate statistically significant difference in columns; different superscript lowercase letters indicate statistically significant difference in rows.

⁣^∗^*t* test; ^*r*^Repeated ANOVA test.

**Table 3:** Comparison of the percentage of successful anesthesia, analgesic intake, and mean age between the light- and dark-eyed patients.

	Light eyed	Dark eyed	*p* value
Successful anesthesia	40/55 patients (72.73%)	37/55 patients (62.27%)	0.567^X^^2^
Analgesic intake	6/55 patients (10.91%)	8/55 patients (14.54%)	0.495^X^^2^
Mean age ± SD (min–max)	34.4 ± 9.9 (20–52)	31.3 ± 9.1 (18–48)	0.132⁣^∗^

Abbreviation: SD, standard deviation; X_Chi−square⁣test_^2^.

⁣^∗^*t* test.

## Data Availability

All data generated or analyzed during this study are included within the article.
